# Acknowledgement to Reviewers of *Life* in 2018

**DOI:** 10.3390/life9010007

**Published:** 2019-01-09

**Authors:** Life Editorial Office

**Affiliations:** MDPI, St. Alban-Anlage 66, 4052 Basel, Switzerland

Rigorous peer-review is the corner-stone of high-quality academic publishing. The editorial team greatly appreciates the reviewers who contributed their knowledge and expertise to the journal’s editorial process over the past 12 months. In 2018, a total of 65 papers were published in the journal, with a median time to first decision of 16 days and a median time to publication of 43 days. The editors would like to express their sincere gratitude to the following reviewers for their cooperation and dedication in 2018:
Altwegg, KathrinLo Giudice, AngelinaAmor, Daniel R.Lücking, RobertAres, SaúlLueth, Virgil W.Arnold, RolandMaldener, IrisBada, JeffMancinelli, Rocco LBains, WilliamMansy, SherefBej, AsimMao, MengBelinky, FridaMarnocha, CassandraBenison, KathleenMassa, GioiaBenner, ChristopherMaurel, Marie-ChristineBernhardt, Harold S.Mayer, ChristianBevilacqua, AntonioMayhew, Lisa E.Billette, ChristopheMichaelian, KaroBlanco, CeliaMirete, SalvadorBoto, LuisMolina-Heredia, Fernando P.Botta, LorenzoMontalvo-Rodríguez, RafaelBouaïcha, NoureddineMontañez Martinez, RaulBrack, AndréMoore, Allen J.Buckel, WolfgangMoran, JosephBurton, Aaron S.Moras, DinoCafferty, BrianMorton, Cynthia M.Carro, LorenaMullineaux, Conrad W.Cavalazzi, BarbaraNa, DokyunChatzitheodoridis, EliasNaraoka, HiroshiChen, IreneNelsen, Matthew P.Cintas, PedroNewman, JeffreyCleaves, Henderson JamesNikoh, NaruoConde-Pueyo, NuriaNorris, VicCorbí, HugoNtougias, SpyridonCordero, RadamesNuevo, MichelCowan, DonOlabarriaga, Silvia D.Damer, BruceOliveira, PauloDanelon, ChristopheOren, AharonDaniel, IsabelleOrlando, Thomas M.De La Varga, HerminiaPaleos, Constantinos M.De Los Santos, Emmanuel LorenzoPangallo, DomenicoDeamer, DavidPascal, RobertDegnan, PatrickPasek, MatthewDelaye, LuisPedreira-Segade, UlysseDelbarre-Ladrat, ChristinePeltzer, AlexanderDeVeaux, Linda C.Perez-Carrasco, RubenDhakal, DipeshPiel, William HD’Hendecourt, LouisPinheiro, VitorDi Giulio, MassimoPinter, TylerDi Mauro, ErnestoPotvin-Trottier, LaurentDixon, NicholasPowner, MatthewDoyle, VinsonPucciarelli, SandraEgel, RichardRagusa, Maria AntoniettaEhira, ShigekiRahm, MartinErastova, ValentinaRanjan, SukritEtxebeste, OierRimmer, PaulFacciotti, Marc T.Robb, CraigFlores García, EnriqueRogers, JeffreyForchhammer, KarlRoss, David S.Fornaro, TeresaRudolph, ChristianFoucher, FrédéricRuiz-Bermejo,Fujihara, AkimasaRuiz-Mirazo, KepaFujii, HodakaRussell, Michael J.Fujita, YuichiSahai, NitaFurukawa, YoshihiroSaitta, Antonino MarcoGómez-Baena, GuadalupeSakamoto, KensakuGonçalves, Luís GafeiraSchirmack, JanoschGoñi-Moreno, AngelSchrenk, MatthewGonzales, Rafael NavarroShain, Daniel H.Gooley, PaulShawn, McGlynnGottlieb, AlexandraShimizu, KazuyukiGrishkan, IsabellaSingh, Nitin KGueidan, CécileSonnino, GiorgioGuespin, JanineŠponer, JiříGutarowska, BeataStano, PasqualeHiggs, PaulStrassmann, JoanHsu, Fang RongStrazewski, PeterHuang, HaoSuccurro, AntonellaJheeta, SohanSun, FengjieJia, TonySurman, Andrew J.Jimenez, M. AngelesTakashima, MasakoJudit, ŠponerTakezaki, NaokoKaddour, HusseinTan, CheemengKalsi, MeghaTessera, MarcKarunakaran, SuneeshThiel, TeresaKerou, MelinaTreu, RolandKiga, DaisukeTrindle, CarlKing, HelenTyystjärvi, TainaKirkendall, LawrenceVallée, YannickKirschner, RolandVan Doninck, KarineKitadai, NorioVaverková, MagdaKohno, NobuakiVavitsas, KonstantinosKolb, VeraVideau, PatrickKooij, Pepijn W.Walde, PeterKun, AdamWieczorek, Rafal M.Kunnev, DimiterWolk, Coleman PeterKwon, YongchanZabel, PaulLebrun, VincentZaritsky, AriehLehman, NilesZentner, GabrielLeuko, StefanZhang, Cheng-CaiLiu, AllenZuo, Ran

